# Integration of eye health into primary care services in Tanzania: a qualitative investigation of experiences in two districts

**DOI:** 10.1186/s12913-017-2787-x

**Published:** 2017-12-13

**Authors:** Emma Jolley, Milka Mafwiri, Joanna Hunter, Elena Schmidt

**Affiliations:** 1 Sightsavers, 35 Perrymount Road, Haywards Heath, UK; 2 Muhimbili University of Health and Allied Sciences, Dar-es-Salaam, Tanzania; 3 University of Leeds, Leeds, UK

**Keywords:** Primary eye care - primary health workers, Health systems - integration

## Abstract

**Background:**

Visual impairment is a public health problem in sub-Saharan Africa, affecting nearly 5% of the population. Efforts to combat avoidable causes have been hampered by weak health systems and little evidence exists to suggest what interventions may be effective to improve the situation. Despite this, there are calls to promote some specific interventions, one of which being the closer integration of eye health services into health systems, often focusing on training primary health workers to deliver basic eye health services. This study seeks to understand how eye health services are delivered by primary health workers who have received training and what constraints remain to effective service provision.

**Methods:**

This was a qualitative investigation into the experiences of 20 primary health workers trained in primary eye care and eight key informants working within specialist eye health services or regional and district health management positions in two districts in Tanzania.

**Results:**

Despite feeling confident in their own eye care skills, most primary health workers felt constrained in the services they could provide to their communities by insufficient resources needed for diagnosis and treatment, and by lack of systematic supportive supervision to their work. Specialist ophthalmic staff were aware of this issue, although for the most part they felt it was not within their capacity to remedy and that it fell within the remit of general health managers. Many participants discussed the low support to eye health from the national government, evidenced through the lack of dedicated funding to the area and traditional reliance on outside funds including international charities.

**Conclusions:**

Although training of primary health workers is useful, it is recognised that is not sufficient to address the burden of eye health disease present in rural communities in Tanzania. It is likely that broader engagement with the general health system, and most likely with the private sector, will be necessary to improve the coverage of eye health care to remote and poor communities such as those in Morogoro. Further investment is needed to develop innovative approaches to delivering eye health services, including preventative, curative and rehabilitative services.

## Background

In 2010 there were an estimated 4.8 million blind people, and 16.6 million people with moderate and severe visual impairment in sub-Saharan Africa (SSA) [1]. This equates to an all-age prevalence in SSA of 1.2 and 3.8% respectively, compared with 0.4 and 2.7% globally, highlighting the disproportionate burden of visual impairment found in SSA compared with other regions. This inequality in prevalence is due to several factors including the endemicity of two blinding neglected tropical diseases (trachoma and onchocerciasis) in many parts of the continent, but to a large extent it exists because a large proportion of people in SSA suffer from preventable or treatable eye health problems that are easily resolved in better resourced parts of the world [2].

In addition to blinding eye conditions, many people in SSA suffer from non-blinding ocular conditions such as trauma, conjunctivitis, and presbyopia [3, 4]. Although non-blinding and often self-limiting, these conditions can cause pain and irritation and potentially impede an individual’s ability to participate fully in society or economic activities. Children in particular are susceptible to eye infections such as conjunctivitis which may be distressing and may cause their parents to seek treatment. A study in Nigeria found that patients with red or itchy eyes were the most likely to visit a hospital for care, whereas people experiencing pain in their eyes were more likely to visit a pharmacist close to their home [4]. The implications of such health seeking behaviour may be that specialist eye health providers may be spending a lot of clinical time on conditions such as red and itchy eyes that could be dealt with by lower health cadres, rather than more potentially serious conditions which require specialist attention.

Many of the problems associated with eye health service provision in SSA have been attributed to low numbers of human resources and unequal distribution of specialist staff within countries. The African human resources for health crisis is well documented as affecting the availability and quality of care, and the eye health sector is not immune from the shortage in adequately trained and motivated staff, deployed to the areas that need them most. [5] A recent study by Palmer et al. found that in SSA only five countries out of 21 included in the review are currently meeting WHO standards for ophthalmic surgical personnel, and that is not expected to change by 2020 [6, 7].

One solution put forward to improve the access of eye health services for people in SSA is to integrate eye health into existing primary health services to deliver primary eye care that would be closer to communities, and be able to provide information as well as quickly refer eye health problems or treat them where possible [8]. The inclusion of primary eye care as a strategy in managing the SSA eye disease burden, alongside the well-publicised strengthening of general primary health systems throughout the continent, has led to an increasing number of primary eye care initiatives throughout the continent. Unfortunately, the evidence surrounding the effectiveness of primary eye care in dealing with eye disease remains weak as few primary eye care initiatives have been well documented, and fewer still have been rigorously evaluated [9–11].

Eye health is seen as a specialist area of healthcare, as many visually impairing conditions, such as cataract or glaucoma, cannot be easily diagnosed and treated in primary care facilities, particularly in resource poor settings. Therefore, the opinions on the added value of primary eye care and the benefits of task shifting in low income countries are often divided. The proponents of the primary eye care approach identify two main pathways through which primary care can contribute to the elimination of avoidable blindness. [9] First, primary health facilities are located closer to and have better interactions with the communities. Therefore, primary health workers (PHWs), if trained and equipped, are in good position to raise awareness about eye diseases and identify and refer patients with latent eye conditions early on to prevent complications. Secondly, PHWs can treat common simple eye conditions, such as conjunctivitis or minor trauma at primary level, and prevent large numbers of patients travelling to distant and often overstretched secondary hospitals saving both patient and provider time and costs. These two propositions however are often thought to be hypothetical, as there is barely any high quality evidence demonstrating positive impact of task shifting on either service delivery or population eye health in low income settings. In addition, there is limited evidence on the process of task shifting or on the opportunities and challenges of integration of eye care tasks within wider primary health systems.

A key objective described on page 2 of the World Health Organisation’s (WHO’s) Global Action Plan on Avoidable Blindness is to *‘encourage the development and implementation of integrated national eye health policies, plans and programmes to enhance universal eye health with activities in line with WHO’s framework for action for strengthening health systems to improve health outcomes’*. [10] In order for this to happen, health care planers need to understand how the existing health system works and how eye health services are delivered within and alongside it. Such analyses are currently underway in multiple countries in SSA using tools developed both by the WHO and other agencies that draw on both secondary and primary data sources to inform their planning and decision making [11, 12]. Such assessment tools often draw on the WHO’s health system ‘building blocks’ framework as a point of reference of how a health system should operate [13]. The WHO describes the six interconnected components, or blocks, of a health system as: Governance and Leadership; Health Care Financing; Health Workforce; Medical Products and Technologies; Information and Research; and Service Delivery.

Efforts to integrate eye care into primary health care to date have mainly focused on the workforce building block, often by providing pre- and in-service training to PHWs on primary eye care and promoting task shifting of eye health service from specialist providers to PHWs although the evidence of the success of these interventions remains inconclusive [9, 14–19]. Very few studies have examined how primary eye care services work in practice, where PHWs are trained to deliver such services.

### Study setting

In 2011, 116 PHWs in two districts in Morogoro region, Tanzania attended a primary eye care training course provided by local and national level eye health specialists. The main objective of the 4 day training course, which has been described in a previously published article [18], was to improve access to basic eye care by early identification, treatment and referral of eye diseases. Three years later a qualitative evaluation of the training was conducted to understand how the training affected PHW practice (reported elsewhere [18]), but also to understand how eye health services were being delivered alongside and within the health system, that is to say, how well integrated primary eye care was into primary health care in these areas.

The objective of this paper is to examine how eye health services are integrated into the primary health system from the perspective of PHWs trained in primary eye care and regional and district health managers. Specifically we wanted to investigate how PHWs who had been trained in eye health are supported to deliver primary eye care services within their communities by other health system components including referral networks, supportive supervision, medical equipment and supplies and health information.

## Methods

This was a cross sectional, qualitative study, using purposive sampling and in-depth interviews to collect the data. Data was collected in June 2013.

The study design and methodology chosen by the researchers was the most appropriate to meet the study’s aims and objectives. A qualitative approach was taken in this study as it was considered the most appropriate way to explore the perspectives of participants and gain an in-depth understanding of primary eye care services. We chose in-depth interviews as the data collection methodology as while they allow the researcher to set the general agenda, they allow for plenty of room for the participant to develop their own account of issues important to them.

### Study area and participants

Study participants included 20 PHWs selected from the total of 116 PHWs trained in primary eye care in 2011 in Morogoro rural and Mvomero districts, Morogoro region, Tanzania.

Purposive sampling was used to select 20 (10 per district) facilities where at least one PHW had been trained in primary eye care in 2011. The facilities were selected to ensure variability in relation to the facility type (dispensary or health centre) and distance from the district and regional hospitals. These attributes were thought to be important because these can determine the types of eye problems presented in the facility, the number and qualifications of staff, and the support received from the wider health system. [15]

Additionally, the study included eight key informants who had specialist knowledge of eye care, in the two districts. These included district and regional health care managers and eye care specialist staff working in district and regional hospitals.

### Research instruments and data collection

A semi-structured topic guide was used to guide a conversation exploring participants’ perceptions of the training and the experiences of application of the newly acquired knowledge and skills in practice. The topic guide allowed the researchers to conduct an open conversation with participants, allowing participants to express themselves fully and discuss issues of interest to them in detail, while ensuring topics of interest to the researchers were covered. The main themes of the topic guide were: 1) the participants’ background, training and experience, 2) their experience in providing eye care services, 3) the training they attended, 4) the impact they consider the training to have had on them and their practice, 5) the equipment and supplies available to them, and 6) how they record data and make referrals. Demographic details were captured in a separate form.

The researchers visited participants in their working stations where interviews took place in private areas. An audio recorder was used to record the interviews, which were transcribed by the researchers as soon as possible following the interviews.

Interview data was supplemented with field notes which recorded interview dynamics and observations of the medical equipment and supplies available in the facilities for primary eye care. Most interviews took place in English but a translator was available during all interviews to assist those who were more comfortable in Swahili.

### Data management and analysis

All interviews were digitally recorded and transcribed verbatim. Where idiom or local names were used we strived to remain faithful to ensure the participants’ meaning was not lost or hidden by our interpretation of their meaning. The interview transcripts were checked for accuracy against the digital recording, and analysed using thematic content analysis [15]. This approach allowed the study team to identify recurring themes and describe the salient issues that emerged from the data. NVivo 10 Software was used to store and analyse the data [16].

The coding of data started with an a priori set of codes developed from the topic guide supplemented by additional codes arising from an initial reading of the transcripts. Further codes were added as the transcripts were analysed in detail and all transcripts were read through a further time to ensure the coding was consistent across the transcripts.

Themes were developed through an iterative process of reading the text and grouping the codes together into categories. The researchers discussed the categories and were guided to some extent by the study aims and research questions, although we prioritised the themes emphasised in the empirical data. For example, the lack of post-training supervision of staff was a major theme emphasised by nearly all informants when asked about the records they keep of patients’ eye conditions. This theme was informed by combining codes such as lack of specialist staff, Health Management Information Systems and finance. Table 1 provides an example of how the themes were developed.

**Table 1:** Example of codes, categories and themes

Theme	Category	Code	Transcribed text
Post-training supervision	Resourcing	Regional finance	“we don’t have the money to pay the staff. We don’t have the money to buy the fuel, to maintain the vehicle. Yes, so you cannot go to the villages.” (key informant)
	Human resource development	Prioritisation of eye health	“Because since we got the eye care training, they did not prioritise it, within the health facilities of management they did not prioritise. Because they didn’t give it priority, we asked at least we could have support, regular supervision. This could help us and update us with information because many people in the communities have many problems to do with the eyes but they do not know.” (Male Clinical Officer)
		Confidence in skills	“I think I can benefit, because if people come here for supervision I will have a chance to explain how I feel about the work, I will have also a chance to explain what I need and maybe I need to know something about this. What if a patient come with this... hmm maybe a patient come with an eye injury so how am I going to manage that person before I, he reach there. So something like that.” (Female Nurse)

The researchers weighed a number of issues when choosing quotes to support the findings in this manuscript. While wishing to preserve the context and credibility provided by the data, we also needed to remain pragmatic about the space limitations and importance of maintaining focus on the stated objectives. The quotes chosen are intended to illustrate the researchers’ interpretation of the data and the analyses that answer the stated research aims.

### Study ethical considerations and trustworthiness

Permissions to conduct the study were sought from the District and Regional health authorities and from the senior management in each facility. Written informed consent was obtained from all participants prior to the interviews. The information and consent forms were made available to participants in both Swahili and English and were discussed with them prior to the interviews starting. Ethics approvals were granted by the Ethical review committees at the Muhimbili University of Health and Allied Sciences (MUHAS), Tanzania, the National Institute for Medical research (NIMR), Tanzania and the University of Leeds, UK.

Data protection was a priority throughout the research process. The recorded interviews were saved onto encrypted data sticks and transcripts were saved onto encrypted files. All participant names were replaced by number codes. The information obtained was used for the purpose of the study only and no one outside the study team had access to the data.

Rigour and trustworthiness in the study were promoted in several ways, which have been detailed throughout this manuscript. In order to ensure valid results we employed appropriate research design and methodology. This included ensuring an appropriate recruitment strategy and a data collection methodology that addressed the research issue as well as abiding by ethical good practice. The data analysis was rigorous and we have presented data within the text in an attempt to promote the transparency of our methods.

## Results

We recruited 20 PHWs 10 male and 10 female, with equal numbers representing two districts. Five (25%) were nurses and 15 were clinical officers with a mean of 5 years in their current position; 18 (90%) worked in dispensaries, while 2 worked in health centres. Table 2 described the characteristics of the primary health care workers.

**Table 2:** Demographic and professional characteristics of the study population (previously published in Mafwiri et al. (2016) [28])

Characteristic		Number (*n* = 20) (%)
Gender	Male	10 (50)
	Female	10 (50)
Working station	Morogoro Rural	10 (50)
	Mvomero	10 (50)
Professional qualification	Nurse	5 (25)
	Clinical Officer	15 (75)
Mean time in current position		5 years
Working facility type	Dispensary	18 (90)
	Health Centre	2 (10)
Has access to:	Visual acuity chart	20 (100)
	Torch	13 (65)
	Training manual	16 (80)
	Tetracycline	8 (40)

We also recruited eight key informants; four each male and female. They had spent a mean of 2 years in their current position; two key informants held positions at the national level while two worked at the regional and four worked at the district levels respectively. Five (63%) were specialist eye health providers. Table 3 described the characteristics of the key informants.

**Table 3:** Characteristics of Key Informant participants (previously published in Mafwiri et al. (2016) [28])

Characteristic		Number (*n* = 8) (%)
Level of informant working station	District	4 (50)
	Regional	2 (25)
	National	2 (25)
Eye health specialist		5 (63)
Gender	Male	4 (50)
	Female	4 (50)
Mean time in current position		2 years^a^

^a^Information only for 5 participants

The majority of participants were satisfied with the training they received and reported feeling more confident in identifying and treating patients with eye conditions. The key informants from the district hospital also noted that since the training the percentage of referrals they received from PHC facilities had grown and the proportion of correct diagnoses of eye diseases by PHC staff had discernibly increased:

*“After the training, I would say about seventy percent are correct diagnoses, about seventy percent are correct. Before that, it was less than ten …” (Key informant).*

However both the training participants and key informants identified a number of barriers to effective application of the newly acquired knowledge and skills within the current PHC system. We divided these barriers into six interrelated themes broadly corresponding to the WHO health system building blocks (see Table 4).

**Table 4:** Summary of study findings

Building Block	Summary findings
Eye health policy and governance	• Responsibility for human resources, equipment and infrastructure necessary to deliver eye care services lies at regional level. • Eye health not perceived as a priority leading to reduced funding.
Health financing	• Health care generally underfunded; • Primary health care financing initiatives such as fee exemptions and community health insurance poorly understood and implemented.
Equipment and supplies	• Inadequate equipment available to provide primary eye care; • Stock-outs of eye medications commonplace at primary health facilities.
Patient records and health service data	• Inadequate data on eye diseases captured within HMIS;
Patient referrals	• No formal referral system in place; • Successful referrals undermined by local poverty levels and low prioritisation of eye health among community.
Post-training supervision of staff	• No regular supervision of primary health workers by eye health specialists; • Supervision to remote facilities undermined by lack of financial resources, however pragmatic solutions could be explored.

### Eye health policy and governance

The first group of issues related to eye health in general rather than specifically to primary eye care. The key informants described different levels of leadership within the eye health system, where the Ministry of Health was responsible for the development and dissemination of eye health policies and the regional authorities were expected to ensure that those policies were translated into practice and understood by district staff. The regions were expected to take responsibility for adequate human resources, equipment and infrastructure necessary to deliver eye care services. The key informants also described the importance of the principles of the continuity of care and the role PHC workers played in the system aimed to improve population eye health:

*“Eye care services are provided within the existing health care delivery system; starting at primary level, which includes the community level where you have community based health workers, frontline health facilities – dispensaries and health centres with health workers oriented on primary eye care …and district hospitals where we have an eye care team – led by an Assistant Medical Officer in Ophthalmology, Ophthalmic Nurse, Optometrist, Ophthalmic Assistant… Secondary level … includes regional referral hospitals where we have an eye care team led by an Ophthalmologist or an Assistant Medical Officer in Ophthalmology. The Highest level of care is tertiary level, where we have four zonal referral hospitals.” (Key informant).*

Although this system was developed as a desired model of care, it was not rolled out throughout the country and the availability of adequately trained cadres at different levels varied widely. One of the key barriers to the establishment of an effective eye care system described by our participants was the lack of perceived urgency in eye health due to the non-lethal nature of eye diseases. As a result eye health policies were of lower priority to the government and limited resources were allocated to eye care programmes. This in turn was the main reason why non-governmental organisations (NGOs) played such a large role in providing eye care across the country; and although all district authorities were obliged to allocate financial resources to eye health during the annual planning cycle, the funds themselves came either from the ‘basket funds’ provided by NGOs or small local grants.

*“The national approach, you know, the government does not give any priority to eye programmes, because eye diseases, they don't kill anybody. The priority is given to malaria, HIV and other diseases. So it’s the Ministry of Health that looks for the sponsors to help …, because the government itself gives very little funding to…eye care.” (Key informant).*

*“According to the Ministry of Health…you have to put some funds for eye problems… they want us to buy equipment … so…basket funds, money [come] from different donors. So the Ministry collects it, then they put it in one basket… [or] they give us local grants; so we can use it too.” (Key informant).*

### Health financing

The lack of financial resources within the Tanzanian health system was described by all to be the reason why health conditions had to be prioritised, and why one group of diseases had to compete with another group for funding. Primary health care, including primary eye care was affected by this general systemic shortage of funds.

In addition, the limited funds available to the system were described as ineffectively and inefficiently used; the processes for funds allocation and disbursement were thought to be lengthy and bureaucratic causing delays with procurement and subsequent shortage of essential medical supplies. A number of interviewees described how these system inefficiencies affected the delivery of services at the primary level. It was explained that in PHC facilities, patients paid fixed fees for access to services which included provisions for medications in stock; this is known as the Community Health Fund (CHF) which in theory facilities can use to supplement the equipment and supplies available to them. Fee exemptions applied to a number of groups including children under five, pregnant women, people with disabilities and people living with HIV However, although individual clinics collect the CHF fees from their patients, they remit the money to the district level and must make a special application to use the funds. This process was not well understood by many PHC workers interviewed, and the key informants described it as bureaucratic and time consuming.

*“There is a shortage of … drugs, of equipment, there is a shortage of staff, probably they lack everything including for eye services. Because there is bureaucracy in getting to buy anything they need using the money they collect in their health facilities. …when they collect that money and send it to their accountant, they are not allowed to take it to purchase something, like medicine…they deposit in their account and it is for the district accountant. And when they want to buy something they need to request …there is a long process… they have to request the district, the district medical health centre, the district executive director…and when … the district director and the accountant are not there, then, there are delays.” (Key informant).*

### Equipment and supplies

It was argued that the shortage of funding in the national healthcare system in general and in eye health affected the availability of equipment and medicines in PHC facilities. The key informants interviewed felt that PHC workers did not have enough equipment to provide adequate levels of eye care in their facilities. Some explained it by the general shortage of equipment in facilities, with eye care facing deficiencies like any other area. Others suggested that eye health was particularly disadvantaged, as it was historically dependent on NGOs and international donors.

*“… since I joined the eye care I never see the government supporting the eye care serves, in terms of equipment and whatever. So most of our eye care services are donor dependent… [although] the roles and responsibilities for purchasing this equipment remain with the government.” (Key informant).*

Primary health workers trained in primary eye care also shared their experiences of accessing medical supplies needed for treating patients with eye care problems. The primary eye care training taught primary health workers to examine eyes using a visual acuity chart and a torch. Most of the respondents felt that these two pieces of equipment were sufficient for carrying out eye examination to the level that they had been taught. All respondents had access to a visual acuity chart in their facilities at the time of the study. Some (13/20 (65%)) had access to torches, although very few still had the torches given to them at the primary eye care training as they had broken. Many used their own torches from home or had personally bought the ones currently in the facility.

*“The torch we got from the training …it’s broken!... It’s rubbish, the light got dim very quickly.” (Female Nurse).*

Some respondents who had been transferred between facilities described having to leave their equipment and information with their previous facility, even in cases when no one there was trained to use it.

*“Yeah, I leave the things there because when you go to a training and you get models or materials it is the property of the centre or facility. So if you transfer to another place, so I leave it to [that] health centre.” (Female Clinical Officer).*

With regards to treatment, study participants explained that medicines for eye health at PHC facilities, which included tetracycline and chloramphenicol eye ointments, were meant to be delivered as part of the general medicine package on a quarterly basis from the Medical Stores Department (MSD), via the District Medical Officer and district pharmacist. However, few facilities visited during the study had both medicines available. The majority did not receive tetracycline as part of their regular deliveries and had been without it for several months. A few primary health workers also mentioned gentamycin (antibacterial alternative to tetracycline) and Vitamin A as important medicines for eye health to which they did not have access. The stock-outs were related largely to shortfalls in funding to the MSD from the government; and primary health workers described a number of strategies they used in their facilities to ensure patients received medicines they needed. These included requesting surplus drugs from the district, reserving medicine for complex cases and children, getting patients to buy the medicine themselves, dipping in to the CHF resources and purchasing the medicines out of their personal money. In some facilities primary health workers used alternative medicines available to them, even when this was different from the treatment protocols they had been taught. For example, chloramphenicol was much more readily available and staff used it as an alternative to tetracycline.

*“The government, they didn’t bring the money to the medical stores department. So it is going to be very difficult for them to distribute the medicines. So, since December in [dispensary] we didn’t receive any kind of medicine. Instead, we are buying through CHF which is a contribution from our people in our catchment areas.” (Male Clinical Officer).*

*“There is a time when we have nothing so I go there and buy one or two [bottles of tetracycline] and put there, so that helps children when they are born…my own money.” (Male Clinical Officer).*

Observations made in the facilities showed that only eight out of 20 primary health workers trained (40%) had tetracycline in their facility at the time of the data collection.

### Patient records and health service data

The management information system to record and report information on eye health in Tanzania health facilities is integrated into the general health management information system (HMIS). Staff in primary health facilities maintain register books of all patients attending for all issues. The registers include personal details, a brief detail of the problem, a category for it to fit in to, and a note on whether the patient was treated, referred or asked to return. At the time of the study there were only three categories for eye diseases in the register: Trachoma, Conjunctivitis and Other. Many primary health workers described this general register as being inadequate for recording information on eye diseases. Some had established their own system of records with extra details on eye problems presented by their patients. Some created hand written copies of the example of a detailed form given to them during the training. There seemed to be some misunderstanding between the training organisers and trainees about whose responsibility it was to provide eye health recording forms to the facilities. Thus, the trainers delivering the primary eye care training described expecting the primary health workers to replicate the example form provided to them in the training manual. Primary health workers’ expectation was that a sufficient number of copies of the form will be provided to all facilities following the training:

*“In this [general register] report you see only two diseases: conjunctivitis and trachoma. So we need to have another one which have many diseases, many eye diseases…In this dispensary, I just perform myself this exercise [eye specific register] and I write eye diseases…For example here there is trauma, I don't have trauma here. Also I have got some cases of presbyopia which I referred.” (Male Clinical Officer).*

There were some discussions of the use of the data collected in the facilities. Primary health workers described how they used the collected data for following up on patients, specific case finding and community education activities.

*“By keeping those notes I can know how the severity of the disease progresses. So I can educate people. So I can know from this month to next month. For example, if I see more patients, I know that there’s a problem and I start health education. And I check the next month how many patients come with eye problems. If the number decreases then I know the education goes well. If the number still increases then give more health education to ensure the numbers decrease.” (Male Clinical Officer).*

It was further explained that summary reports prepared by primary health workers were sent to the district health management team (DHMT) on a monthly and quarterly basis, where supervising staff was expected to read and investigate any problems identified through the report. District level staff was expected to report this information to the regional eye coordinator and use it for the purpose of monitoring and planning outreach activities. Regional specialist staff received summarised data twice throughout the year, unless they specifically requested an update. They were expected to use the data for advocating resources within their budget and other departmental budgets. Although there was a general agreement among our participants that health information was essential for effective planning for eye care services, many were unsure about the completeness of information available at different levels of decision-making or the effectiveness of its use in practice. For example, some district level officials reported not having seen any reports on eye health from primary facilities, despite this being an important source of information for their work.

### Patient referrals

Although continuity of care was emphasised by all study participants, the implementation of this principle in practice was challenging, and at least two issues in the system of patient referrals were identified during the interviews. First, a number of PHC workers noted that the mechanisms for referring patients between primary and secondary level facilities were not standardized; different PHC facilities used different methods of referral and different documentation. Although a standardized referral form was designed during the primary eye care training, PHC staff had only one copy given to them as part of the training manual and their facilities had no means to photocopy it for all their patients.

*“That referral form was developed … during the training. We developed the first draft and had the first batch at the training… [But] it is difficult to produce enough copies for referral.” (Key informant).*

Another problem identified by both primary health workers and district staff was uptake of referrals. It was pointed out that many patients particularly those in remote areas did not have money to travel to town and couldn’t afford to take a day or more off work to make the trip. In many cases the costs of the specialist eye care, including medications and spectacles, were unaffordable for the patients. This was frustrating and demotivating for primary health workers who made the referral, as despite their best intentions and efforts patients could not benefit from care and their condition continued deteriorating.

*“There are patients who are ready for referral and they [primary health workers] are doing the referrals but they [patients] are not reaching the regional hospital simply because there is no vehicle for them to come here and they don’t have money to use the public transport.” (Key informant).*

It was also argued that the capacity of the specialist services at the regional level were also limited; and in the absence of a regional ophthalmologist, complex and paediatric cases were referred to tertiary care in Dar es Salaam, which created further burden for patients and their households and often resulted in non-uptake of treatment.

### Post-training supervision of staff

Another set of challenges described by our participants related to the lack of follow up and supervision of PHC staff in the period following the training. Nearly all primary health workers described supervision as an essential element of effective primary eye care and wanted to have regular supervision visits by specialist staff to their facilities. It was argued that regular supervision would give primary health workers an opportunity to discuss difficult cases and update their knowledge on eye diseases and medications. District level specialists could also benefit from these visits, as they would learn more about eye conditions and eye care seeking behaviour prevalent in the local communities.

Although the system of general supervision of PHC facilities was well established with quarterly supervision visits by the DHMT, supervision on issues relevant to eye health was not integrated within this system. There was no clear understanding of how eye health supervision should operate or who is responsible for supervisions, either at the district or regional level. Many primary health workers had no interactions with district level specialist staff; for others the interactions were rare and sporadic. The standard forms used by the general supervision teams did not contain any questions pertaining to eye health and focused largely on administrative issues and medical supplies rather than clinical work. PHC workers described feeling isolated from speciality knowledge and were concerned that no one was checking their work to prevent mistakes:

*“Imagine in these 2 years there is no supervision.” (Male Clinical Officer).*

*“There is a general supervision, but what I know … is that no one pays attention to those who were trained in eyes.” (Key informant).*

*“The most pressing issue is the supervision. You see, when you train someone and leave them in the village …without any supportive supervision … I don’t think it’s a good thing…” (Key informant).*

Where supervision on eye specific issues happened, it was opportunistic, sometimes when the general supervisory team happened to include someone with specialist knowledge of eye care, sometimes when a PHC worker knew an eye specialist in the district hospital and took it upon themselves to contact them for an advice. Some PHC workers consulted hospital providers when the latter came to do outreach clinics, or were part of the team which conducted general supervision visits. Occasionally the specialist providers would give the primary health workers feedback on their referrals, but this was also sporadic and largely informal.

*“We have not … managed to go to the health facilities to see how they keep their data and information simply because it was not in the plans. I managed to visit only …few health facilities to see what they are doing. But it was not through the eye care programme. It is only that…I am also a member of the Regional Health Management Team… the team [that] overseas all the health services in the region. So as part of that supervision whenever I reach the health facility, because I am more concerned with the eye, I just go ‘Oh, were you trained in eye care?’ ‘Yes’ ‘What are you doing?’ Look at the registers, ask questions.” (Key informant).*

Furthermore, the composition of the DHMTs responsible for PHC supervision was not tailored to the skills and services available within the PHC facilities they visited. As a result the teams sent to the PHC faculties with the staff trained in eye care often lacked specialist workers able to provide supervisory support. Other teams however included eye care personnel who acted in their capacity of a district supervisor but there was no guarantee that the facilities they visited were staffed by someone trained in primary eye care.

One suggestion put forward was for all supervising staff to be given a basic training on eye health, to enable them to more effectively integrate eye health into their general supervisory visits. In addition to this it would be necessary to adapt the supervision forms to include a component on eye care, to ensure that general supervisions can look for the important indicators of a quality eye health provision at the primary facilities.

*“When they [the district health management team] want to supervise the eyes this man should be in that team, so that when they are doing the supervision they ask all questions, what is the deficiency? What problems you are facing? Then they put it in writing … [and] take it back to the district level.” (Male Clinical Officer).*

Specialist eye care staff in secondary facilities said that they had staff and could potentially organise supervision of PHC staff by specialist eye health workers. They however did not have financial resources for the petrol and per diems to travel to the villages.

*“… we have vehicles, we have vehicles for eye care…. We have a number of staff, adequate staff …at least the ophthalmic nurses, the cataract surgeons …We don’t have money to pay the staff, we don’t have money to buy the fuel, to maintain the vehicle. Yes, so you cannot go to the villages.” (Key informant).*

Some district level officials argued that the supervision of primary health workers on matters of eye health contrasted sharply with the supervision on other health issues, for example HIV, where there was a separate supervision structure for PHC, including a separate supervision form and financing for staff to regularly travel to all facilities. However, significant resources available for this type of supervision were often provided by an external donor and continued only for the duration of the project, after which its future in many cases was uncertain.

## Discussion

This study highlights the interconnectedness of health system building blocks impacting on the eye health service delivery in Tanzania. The application of the new knowledge and skills acquired during a short, focused training by primary health workers was not an easy process. The study identified a number of challenges in the integration of eye care activities into general primary health systems; these barriers related to the wider health system environment; general eye health system; and primary eye care specifically.

The general shortage of resources within the wider health system in Tanzania resulted in the lack of staff, equipment and medical supplies available to eye health and led to a situation where eye health had to compete with other health areas and interventions. The non-lethal nature of eye diseases and historical dependence of eye care services on external donors resulted in low priority given by the government to eye health infrastructure and financing. As a result, many primary health workers trained in eye health had limited opportunities for application of their knowledge in practice because they did not have the required examination equipment and medicines. In addition the use of the scarce resources available to the system was not thought to be particularly efficient, as the process of obtaining funds for purchasing medical supplies was long, cumbersome and bureaucratic.

The training of primary health workers itself appeared to be isolated from other aspects of the local health system. For example, there was no agreed and clearly defined system of supervision of the trained workers; the HMIS system was not adapted to collect information on a full spectrum of eye conditions treated in PHC facilities; the referral systems were not adequate to ensure continuity of care between primary and secondary level facilities, and many patients were not able to uptake the referral due to high patient costs. As a result, although generally satisfied with the primary eye care training provided, many PHC workers felt abandoned and demotivated and they had to find their own opportunistic ways to address the deficiencies within the system, which was both difficult and frustrating.

Ocular morbidity studies from elsewhere in East Africa suggest that around one in six individuals may be suffering from an ocular morbidity at any one time [3]. Even allowing for a number of these being self-limiting, this suggests that ocular morbidities, including blinding and non-blinding conditions, may pose a significant public health issue that is not yet being adequately addressed.

Hypothetically, there are two main pathways through which training primary care workers can lead to the improved population eye health. First, being located close to the communities, trained primary health workers can identify latent eye conditions and refer patients to the upper level facilities for treatment early on, thus preventing complications. Secondly, primary health workers can treat simple eye conditions at primary level, and prevent large numbers of patients attending overstretched secondary hospitals saving patient costs and freeing up capacity of ophthalmic specialist staff to address more complex eye diseases. The findings of this study show that neither pathway is straightforward and both depend on specific systemic changes that need to be put in place and effectively implemented. Thus to be able to diagnose and treat eye conditions at the primary level, primary health workers would require effective systems of ongoing supervision; improved equipment and pharmaceutical supply chains, and improved management information systems for recording and reporting data. All of these would need to be integrated within the wider health system rather than operate alongside it.

The system of referral would also need to be considered. Our findings suggest that mobilisation of primary health workers is likely to lead to an increased case finding and increased number of referrals. However, whether these referrals will result in a higher number of treatments and reduced levels of visual impairment is less certain. In line with other research in low-income countries [20–23], this study shows that the uptake of referrals in such settings is often impeded by financial and logistical barriers, such as high user fees, long distances to facilities and lack of transport. The capacity of specialist services to address an increase in case presenting is also critical, as the shortage of eye care resources and particularly ophthalmic staff in Tanzania and similar settings is a well-known challenge [6, 7]. This means that many referred patients, even if they manage to reach secondary facilities, risk to be left without treatment they need. In the absence of adequate and affordable service at the secondary level, a future increase in demand from primary care could be considered unethical.

In this study we did not specifically explore the capacity of primary care facilities to deliver additional services. The primary care workers interviewed were enthusiastic about being able to provide a better standard of care within their communities and many appeared keen to provide even more services. However, the issues of significant pressure placed on primary care workers and overcrowded primary care facilities have been raised in many studies [24–26]. Therefore primary health workers’ workload, facility space and other resources of the local primary health care systems also need to be considered, when training of health care workers in primary eye care is being planned.

Although the need for significant investment in eye health at all levels of the health system has been repeatedly noted as important [27], it is also important to be realistic about the financial constraints facing Ministries of Health in resource poor settings such as Tanzania. As well as searching for more money for eye health in such settings, one need to look for ways to be more efficient with existing resources and to explore opportunities to include eye health into other existing initiatives that will allow the system to maximise impact at minimal cost.

Although the study highlights many interesting findings about primary eye care in Tanzania, there are a number of limitations that must be acknowledged. Although this study sought to obtain a broad range of insights from the study population, by purposively sampling only a small proportion of those who participated in the training, there may remain further diversity of opinion within the broader group, which is not represented here. Although the study group included several Tanzanians, available time and expertise meant that data collection was conducted by UK based researchers, which may have affected some of the responses provided by the participants in a particular way that may have biased some of the conversations that took place. Despite full information provided at the beginning of the interview, the research may have been perceived as linked to further training or provision of financial or in-kind support.

## Conclusions

Although this is a small scale qualitative study implemented in two districts of one region in Tanzania, the findings raise a number of important questions and policy implications. The study shows that the training of lower level cadres in eye care per se does not necessarily result in improved access to services and better treatment opportunities for patients. Weaknesses within broader health care systems will influence the provision of services such as eye health, no matter how discrete they may seem. For training to be effective, other components of the health system need to be well understood and weaknesses addressed where possible. In this particular situation in rural Tanzania, there is a need for (i) clear lines of responsibility and adequate resources for staff supervision, (ii) effective, uninterrupted systems of procurement of equipment and medicines required by primary eye care; (iii) effective mechanisms for pooling and disbursement of health financing at the provider and patient levels; (iv) appropriate information and patient record systems integrated within the wider HMIS; and (v) effective referral mechanisms, which ensure patient access to care and provider feedback. In this setting in Tanzania, and other settings where similar primary eye care interventions may be occurring there is also a clear need for rigorous research which collects objective treatment and referral information and assesses the impact of task shifting on the availability and access to services.

## Abbreviations

CHF: Community Health Fund
DHMT: District Health Management Team
HIV: Human Immunodeficiency Virus
HMIS: Health Management Information System
MSD: Medical Stores Department
MUHAS: Muhimbili University of Health and Allied Sciences
NGO: Non-Governmental Organisation
NIMR: National Institute for Medical Research
PHC: Primary Health Care
SSA: Sub-Saharan Africa
WHO: World Health Organisation
